# On Syphilitic Meningitis and Sypilitic Cerebral Disease

**Published:** 1874-11

**Authors:** 


### On Syphilitic Meningitis and Syphilitic Cerebral Disease.

In making this valuable contribution to the pathology of syphilis of the nervous tissues. Dr. Bruberger, (Virchow's *Archive*, May 6, 1874), narrates with extreme minuteness the clinical history and *post-mortem* examination of a patient who contracted syphilis in 1874, had destructive ulceration of the soft palate two years later, and sudden complete motor paralysis of the arms and legs occurring while under excitement partly alcoholic, partly of another kind. The paralysis was so complete that the patient was unable to move a finger or toe, or make the slightest effort to change his position in bed. Meanwhile, the cutaneouss ensibility of the body and limbs was ascertained by many careful experiments to be only very slightly diminished, and the susceptibility of the muscles to electrical stimulus was as keen as in health. The muscles of the face and of respiration were not paralyzed, nor was control of the sphincters wholly lost. Consciousness was lost only for a few moments, and having returned, remained unimpaired to the last. The intellect was also intact. Iodide of potassium, in large doses, was administered for four weeks, and then changed for mercurial inunction. Six weeks after admission the patient regained the power of slowly clenching the fist and opening the hand again, but no other improvement ensued. After lingering nearly four months he sank, exhausted by bed-sores and vesical irritation. In making the diagnosis, no hypothesis explained all the symptoms. The bilateral symmetry of the motor palsy and the unimpaired sensorium suggested the cord to be the part attacked. The suddenness of onset indicated extensive hemorrhage or plugging of a large artery, were it not that in syphilis paralysis does occur suddenly without either of these conditions being found after death. Again, a thrombosis or hemorrhage would have almost certainly produced some hemiplegia. Local softening or the presence of a gumma in the medullary tissue was, like hemorrhage, precluded by the undiminished intellectual power. Lastly, disease of the meninges at the base, a frequent form of cranial syphilis, was rendered improbable by the absence of periphiral irritation of all kinds; neither convulsions nor slowness of pulse, nor sinking in of the abdomen, etc., were present. But the *post-mortem* examination revealed most unexpected conditions, the principal being inflammatory thickening of the dura and pia mater in the cervical portion of the spinal cord and base of the brain, producing a leathery sheath which had formed numerous attachments to the medulla and to the bony surfaces. There were also hemorrhages into the spinal cord, with atrophy of the gray substance and considerable widening of the central canal. In the skull, inflammation had spread widely through the meninges over the basilar bone, and had changed the pia mater at the base of the brain into a thick, gray, glue-like mass. Another striking alteration was a peculiar change in the vessels of the skull, causing thickening of their walls and a nodular condition of their calibre; the arteries throughout the rest of the body being free from perceptible change. The remaining phenomena had nothing specially syphilitic. The author then recapitulates the points of interest in the case, and has collected the published records os syphilitic disease in arteries, which consist mainly of the observations of Hughlings Jackson, Moxon, and Clifford Allbutt, and of one or two others. These observations show that ordinary atheroma, if at all a syphilitic product, is not the usual arterial affection, which consists of irregular thickening of the coats, especially of the middle tunic of the vessel, producing nodular projections with narrowing and some loss of elasticity of the vessel, but no fatty or calcareous degeneration of its walls; further, that this inflammatory change of the arteries is very local, attacking the vessels of one or two regions, and not spreading through the arterial system generally. After remarking that the extensive disease of the meninges caused no convulsive affection of any kind, again a peculiarity of syphiltic meningeal disease, the author notes, without offering an explanation, the curious fact that sensation was intact throughout the body, and yet the gray matter of the cord was far advanced in atrophy, while the white fibres were so tightly strangled at one point of the medulla as to produce almost total loss of voluntary muscular power.—*London Med. Record*, July, 8, 1874.—*Monthly Abstract.*

